# Supplementary material for: Design and Synthesis of New Modified Flexible Purine Bases as Potential Inhibitors of Human PNP
Source: Molecules. 2023 Jan 17;28(3):928. doi: 10.3390/molecules28030928 (PMC9921493; doi:10.3390/molecules28030928)

Supplementary Materials

Design and synthesis of new modified flexible purine bases as potential inhibitors of Human PNP

Anastasia Khandazhinskaya[1], Ilja Fateev[2], Anna Maslova[1], Barbara Eletskaya[2], Irina Konstantinova[2], Katerine Seley-Radtke[3], Sergey Kochetkov[1], Elena Matyugina[1]

[1]Engelhardt Institute of Molecular Biology of the Russian Academy of Sciences, 32 Vavilov St., Moscow 119991, Russia

[2]Shemyakin-Ovchinnikov Institute of Bioorganic Chemistry, Russian Academy of Sciences, Moscow 117997, Russia

[3] Department of Chemistry & Biochemistry, University of Maryland, Baltimore County, Baltimore, MD, USA

Content

^{1}H-NMR and ^{13}C-NMR spectra (300 MHz, $CDCl_3$) of target products**.**

HRMS spectra of compounds target products.

Figure S1: Lineweaver–Burk plots for the inosine phosphorolysis at concentrations of compounds 1, 2, 3 and 4 equal to 0, 0.5, 1 and 2 mM. (A) compound 1, (B) compound 2, (C) compound 3, (D) compound 4.

Figure S2: 2D diagrams of complexes PNP with various type of ligands.

Figure S3: Structure of the 3INY with re-docked 7-deazaguanine (An overlap the red wire – native ligand from PDB database; the red stick docked ligand. The box for docking ligands was highlighted in green (12 Å × 10 Å × 10 Å.)

^{1}H-NMR spectrum (300 MHz, DMSO-d6) of compound **1**

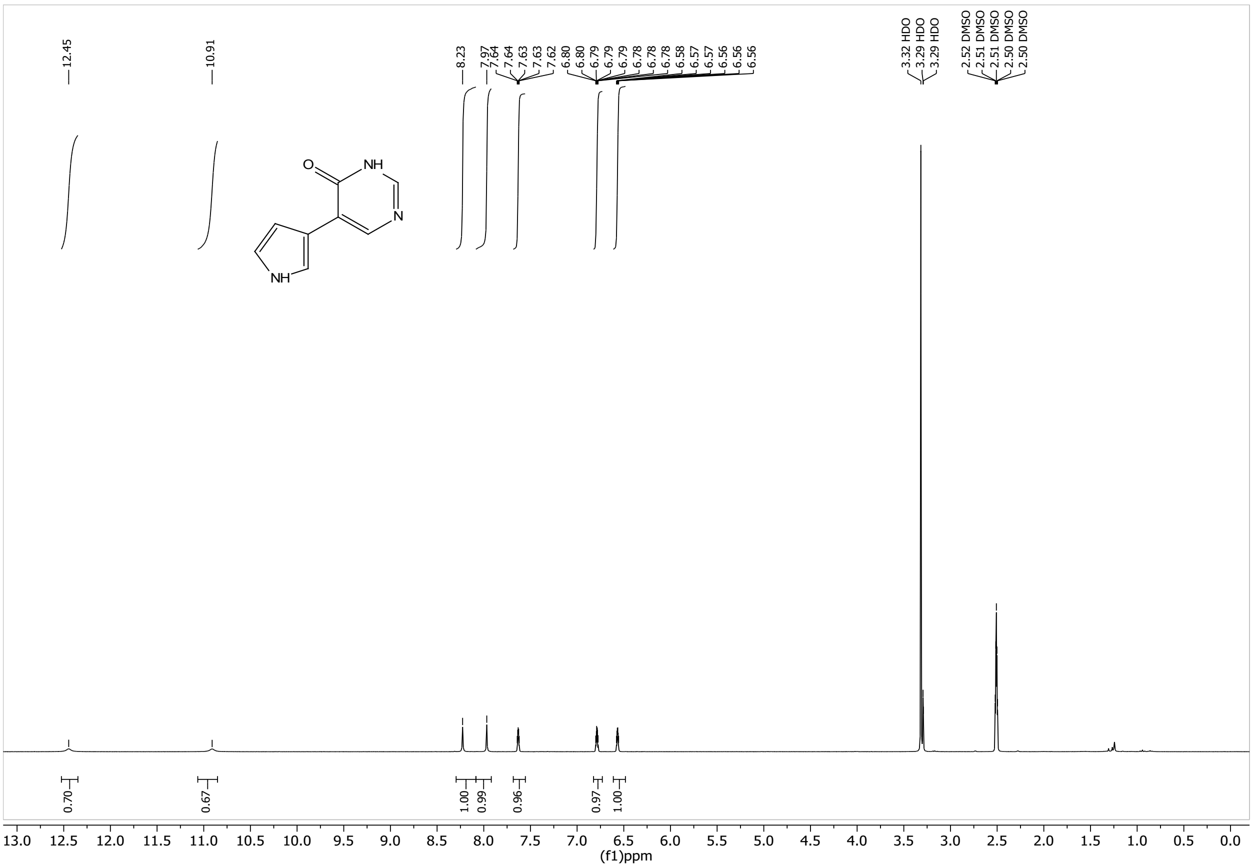

^{13}C-NMR spectrum (75.5 MHz, DMSO-d6) of compound **1**

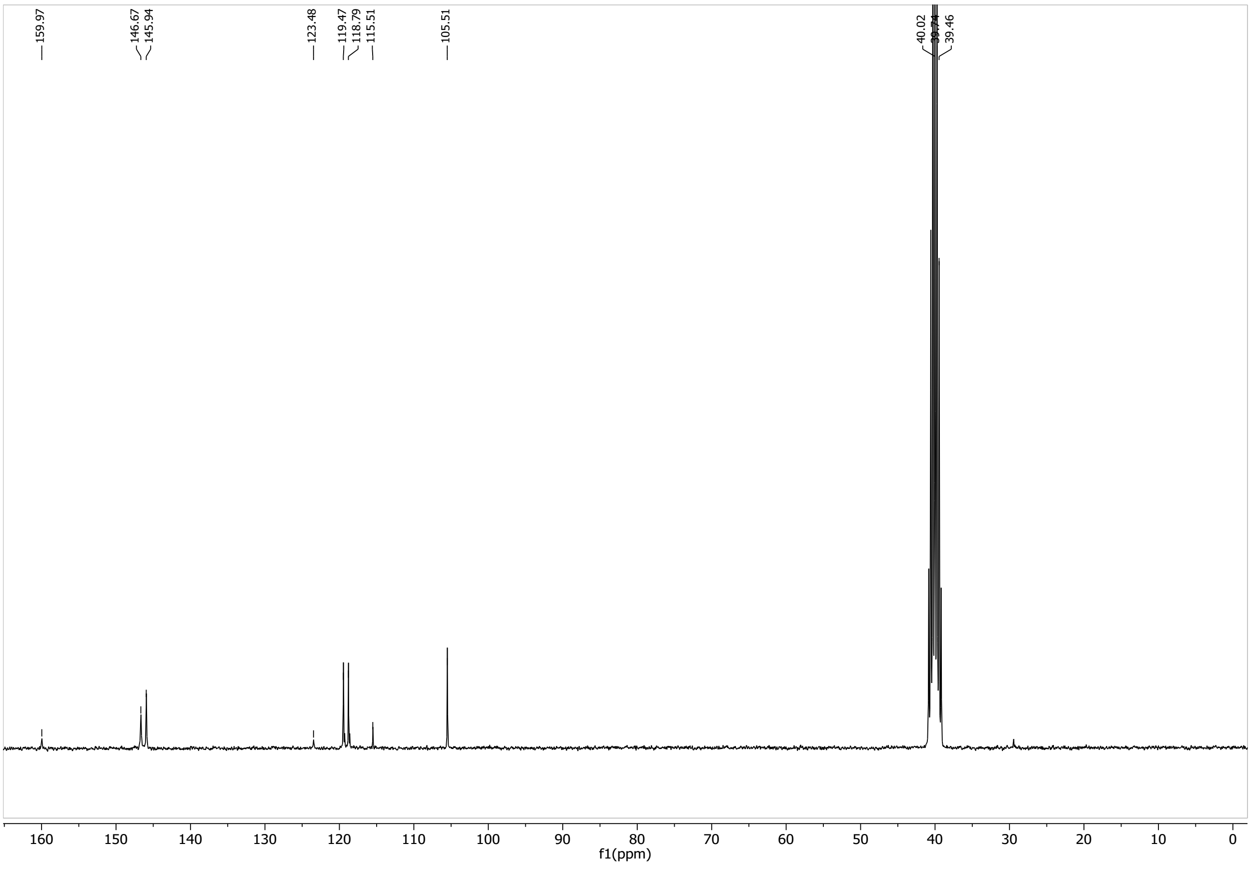

^{1}H-NMR spectrum (300 MHz, CD_3OD) of compound **2**

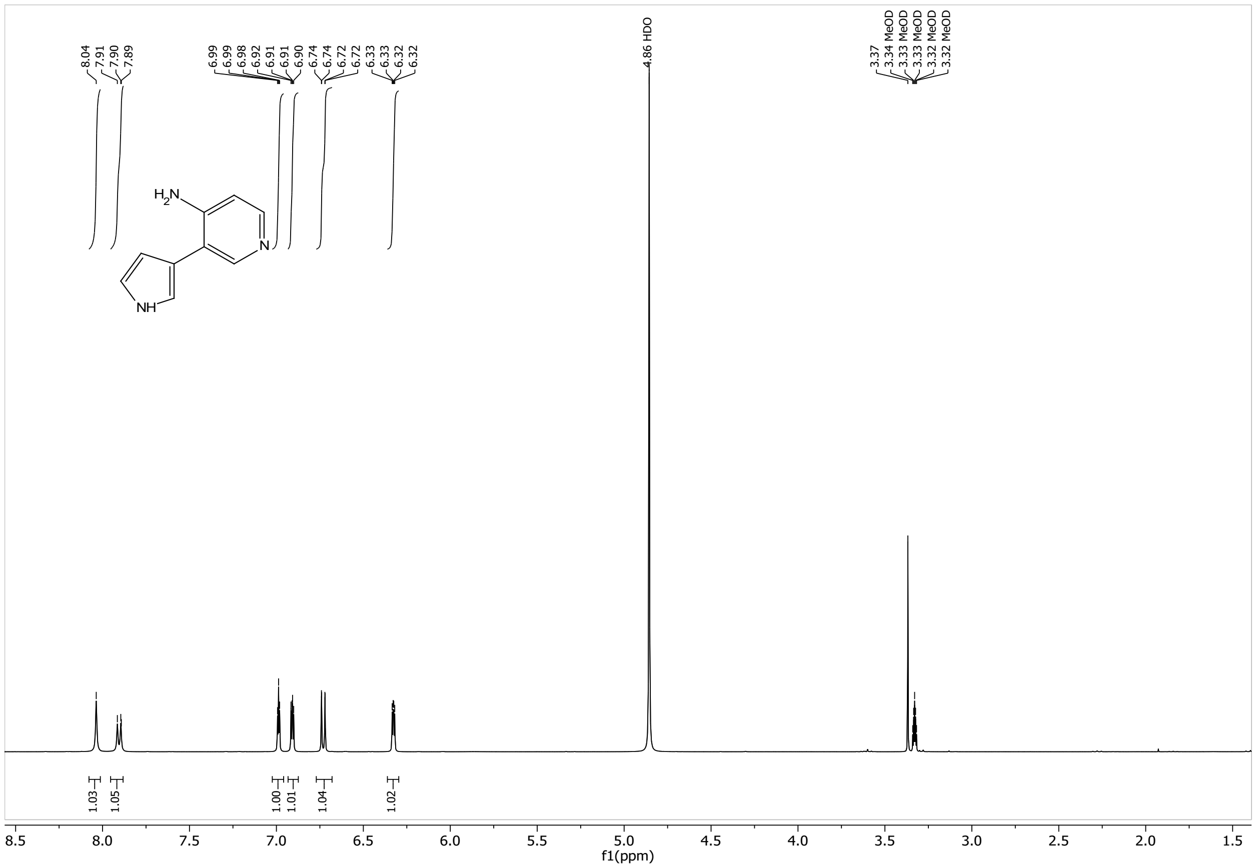

^{13}C-NMR spectrum (75.5 MHz, CD_3OD) of compound **2**

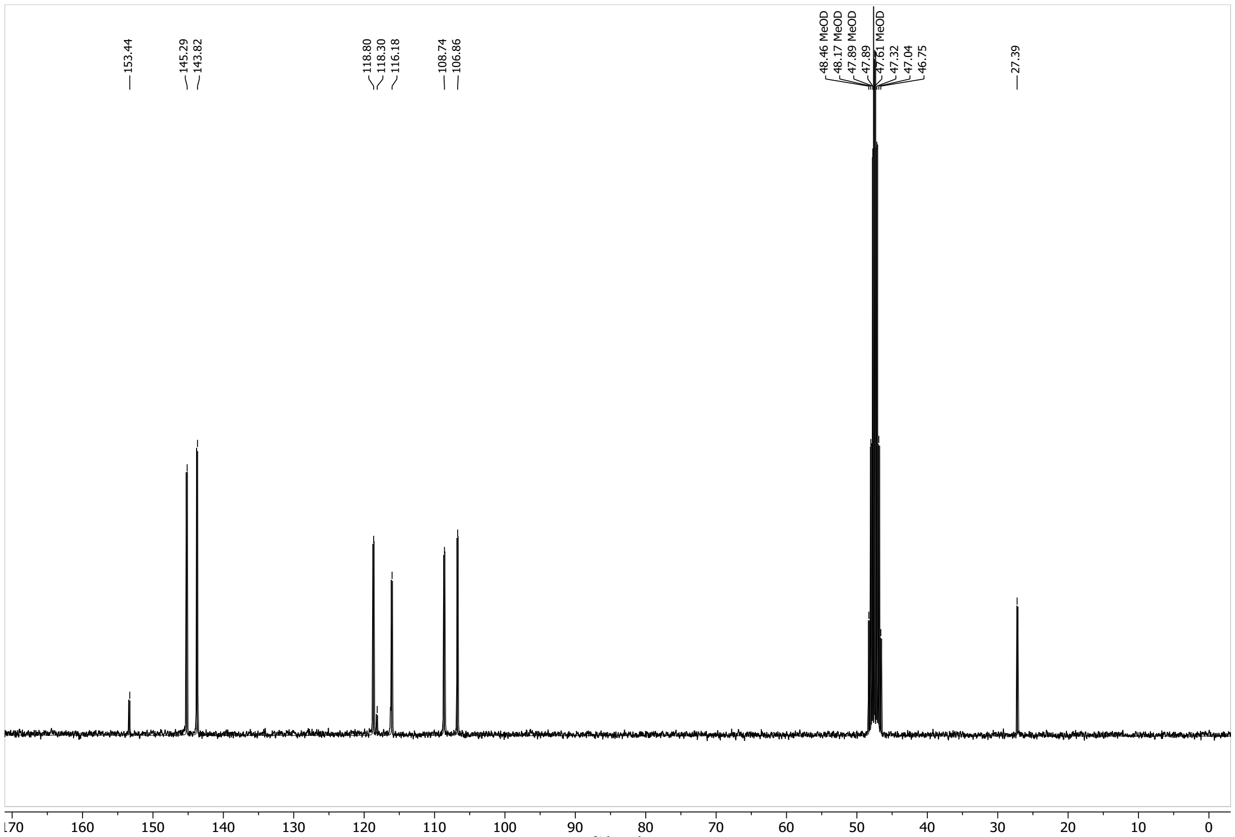

^{1}H-NMR spectrum (300 MHz, DMSO-d6) of compound **3**

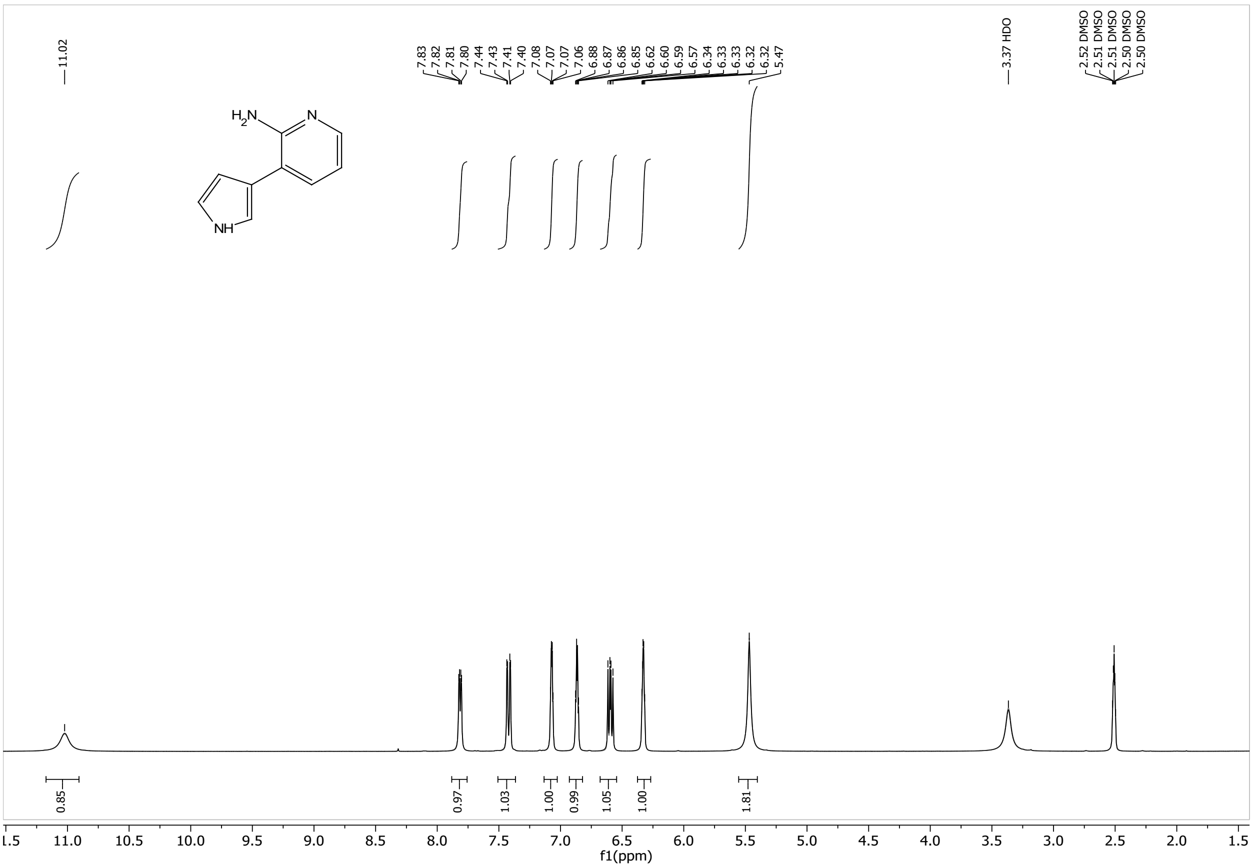

^{13}C-NMR spectrum (75.5 MHz, DMSO-d6) of compound **3**

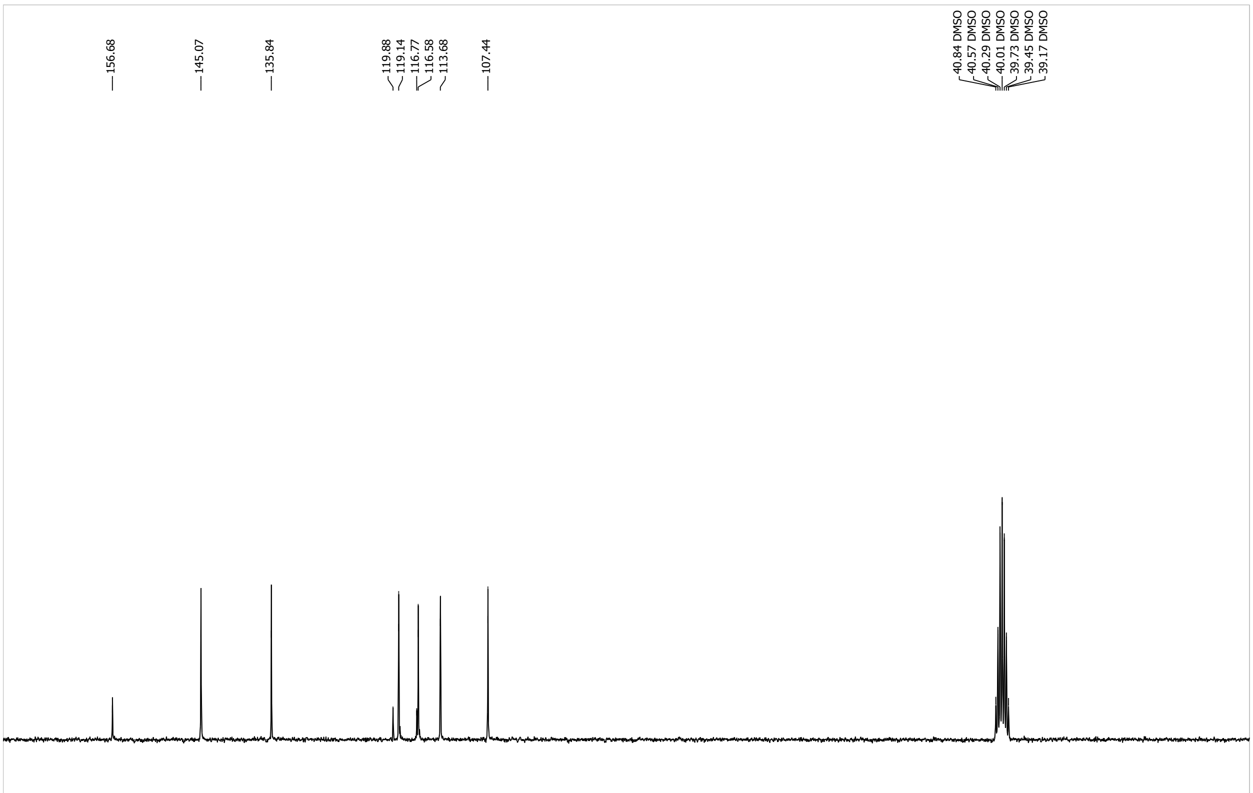

^{1}H-NMR spectrum (300 MHz, $CDCl_3$:CD_3OD) of compound **4**

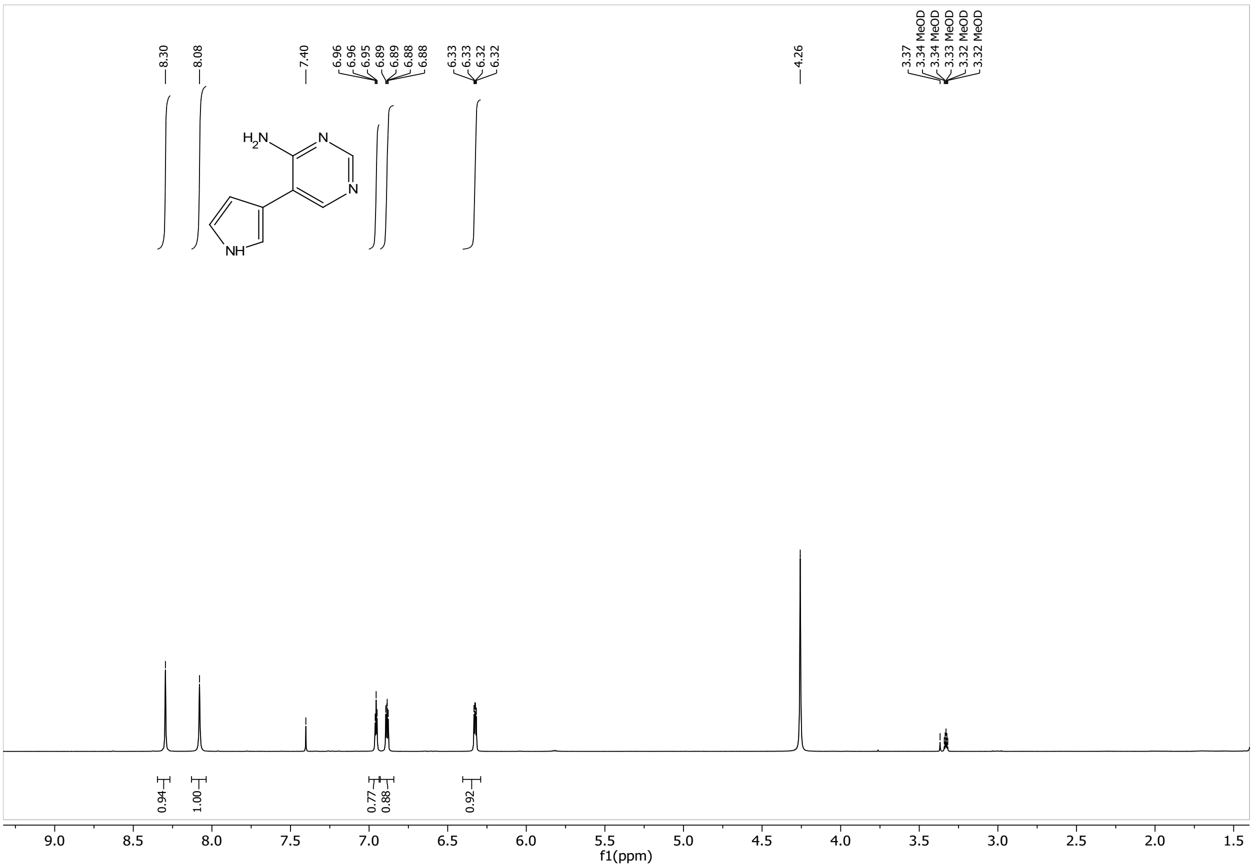

^{13}C-NMR spectrum (75.5 MHz, $CDCl_3$:CD_3OD) of compound **4**

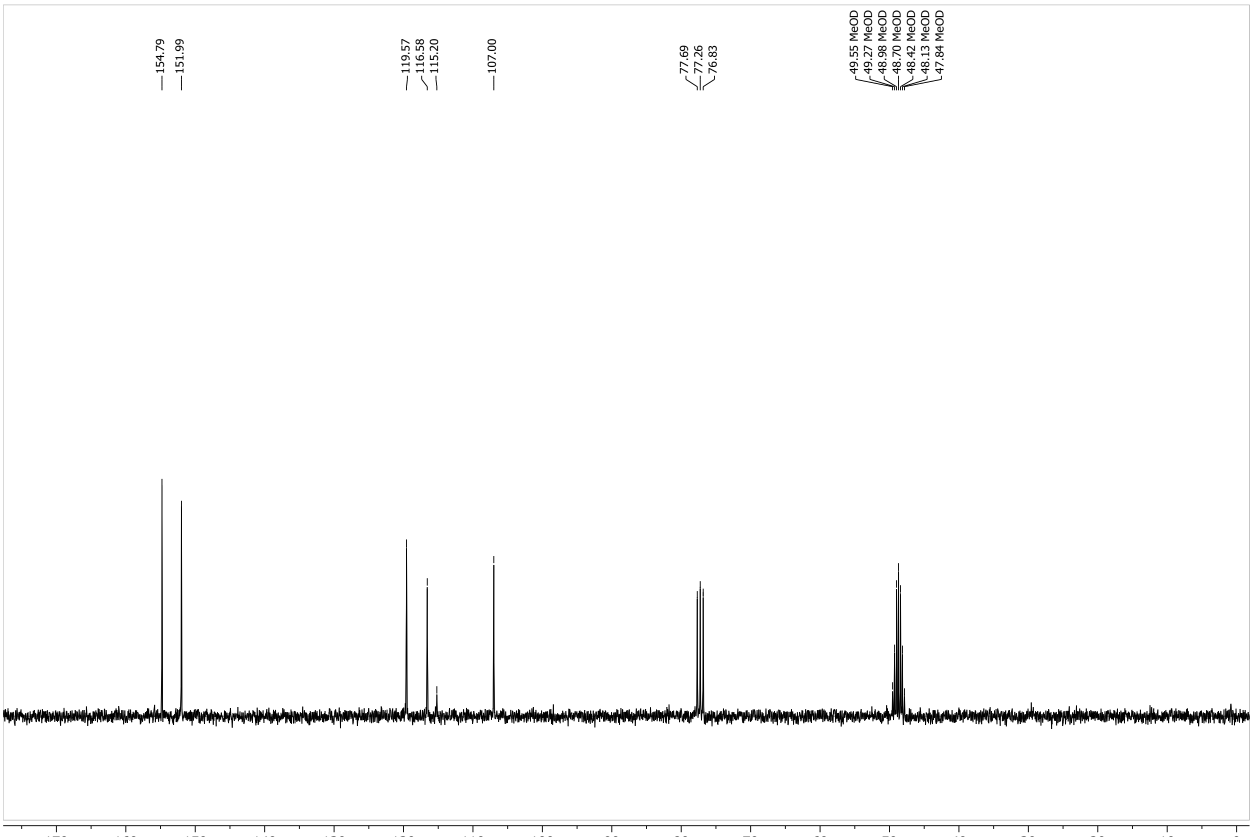

^{1}H-NMR spectrum (300 MHz, CD_3OD) of compound **13**

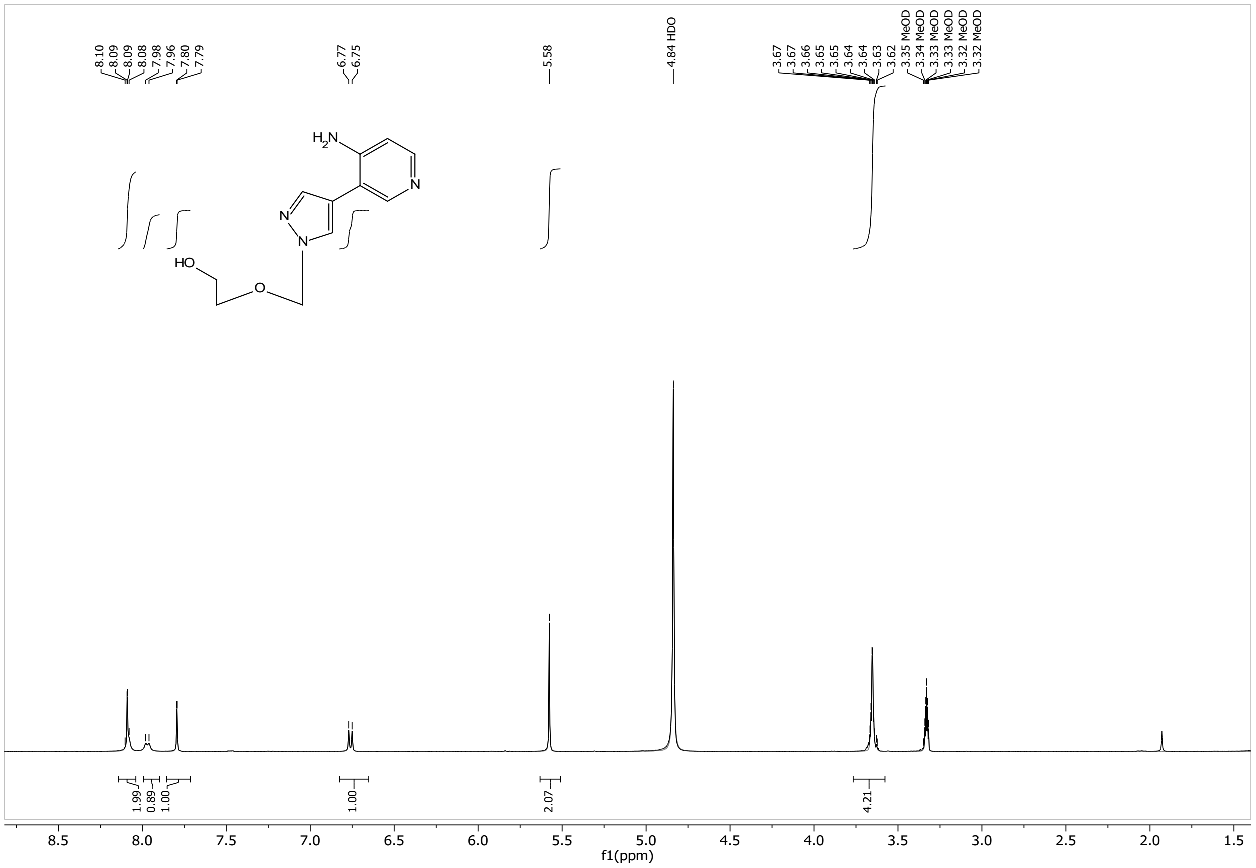

^{13}C-NMR spectrum (75.5 MHz, CD_3OD) of compound **13**

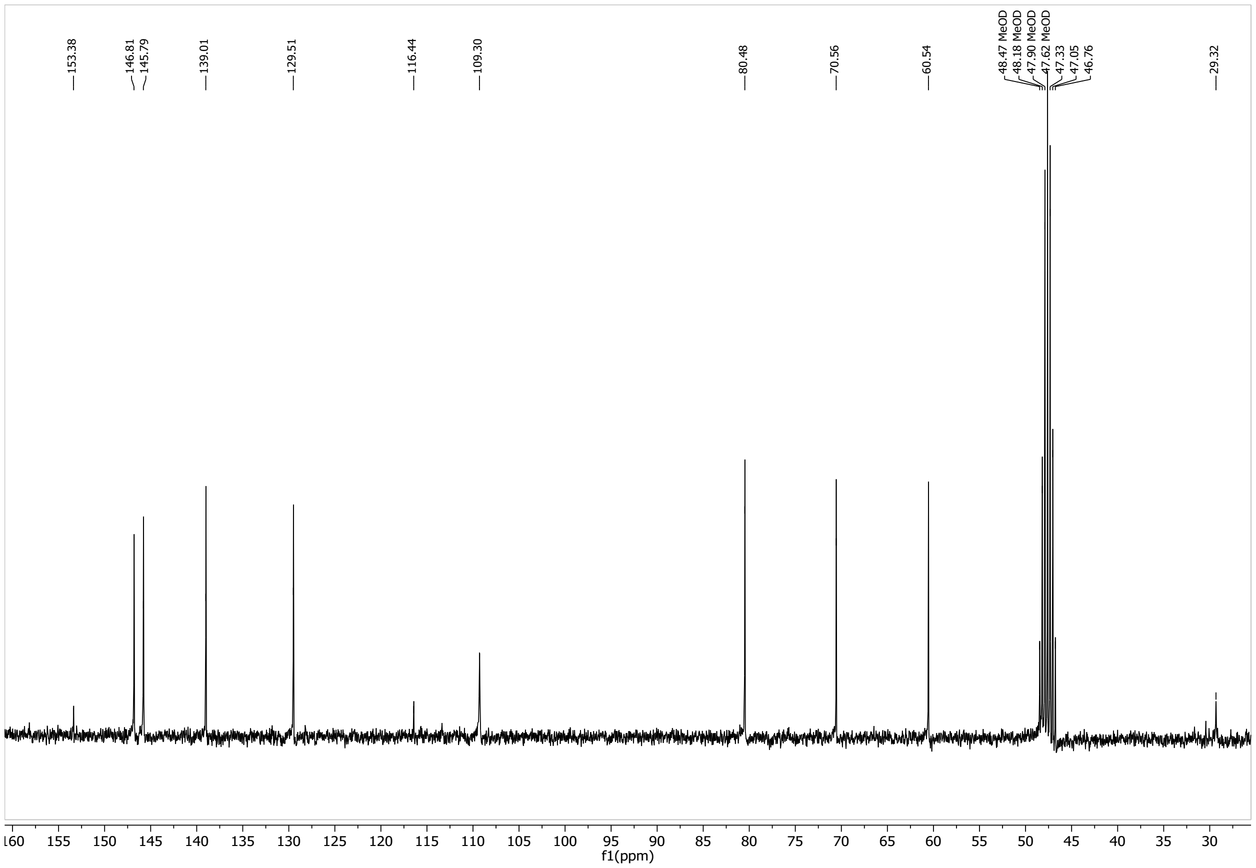

^{1}H-NMR spectrum (300 MHz, CD_3OD) of compound **14**

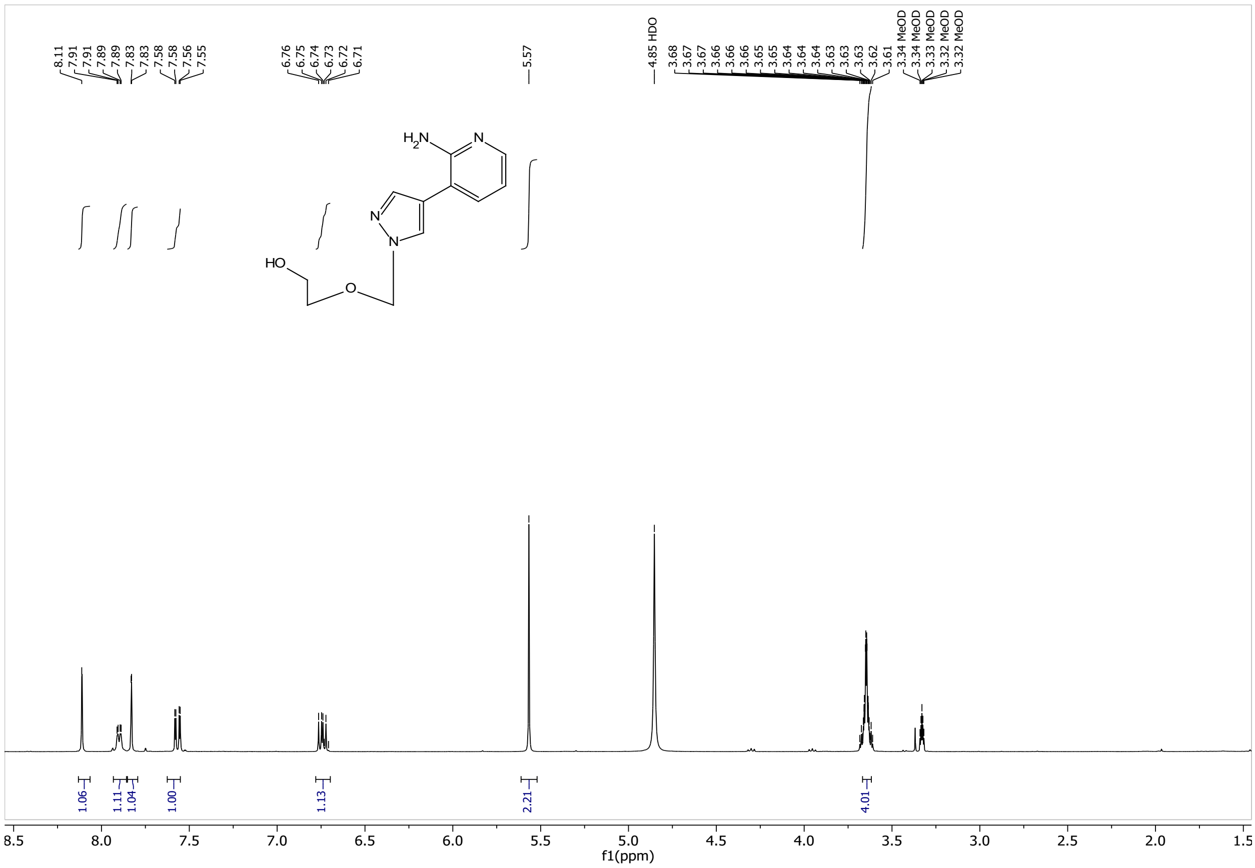

^{13}C-NMR spectrum (75.5 MHz, CD_3OD) of compound **14**

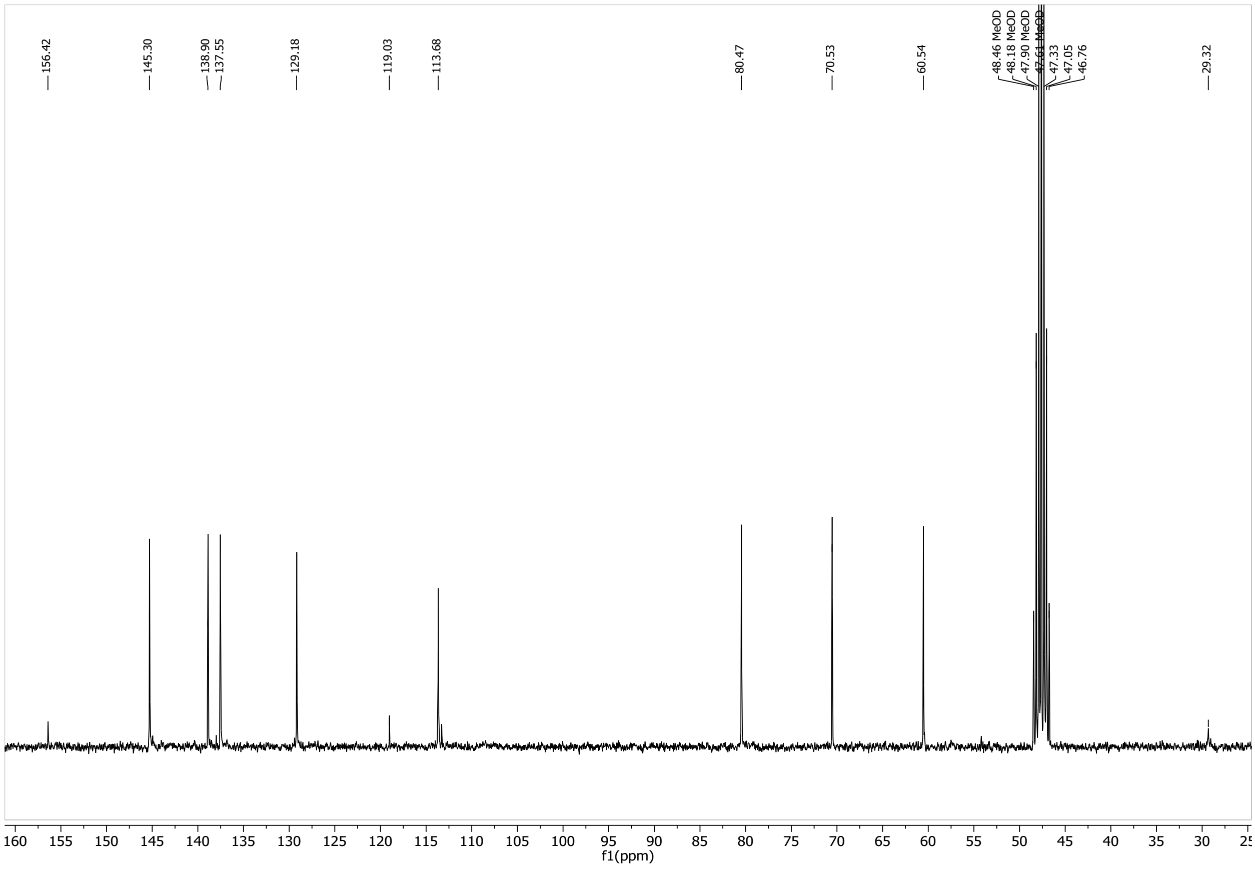

^{1}H-NMR spectrum (300 MHz, CD_3OD) of compound **15**

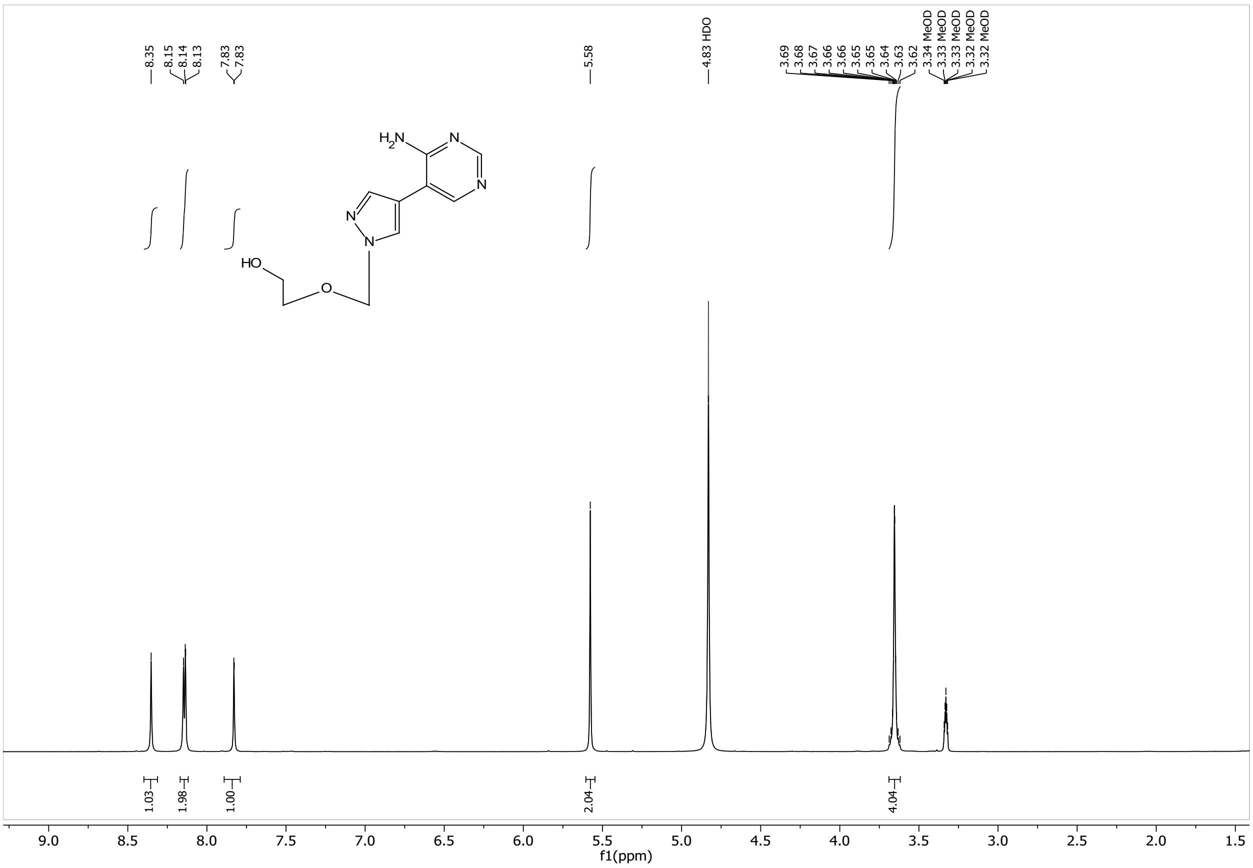

^{13}C-NMR spectrum (75.5 MHz, CD_3OD) of compound **15**

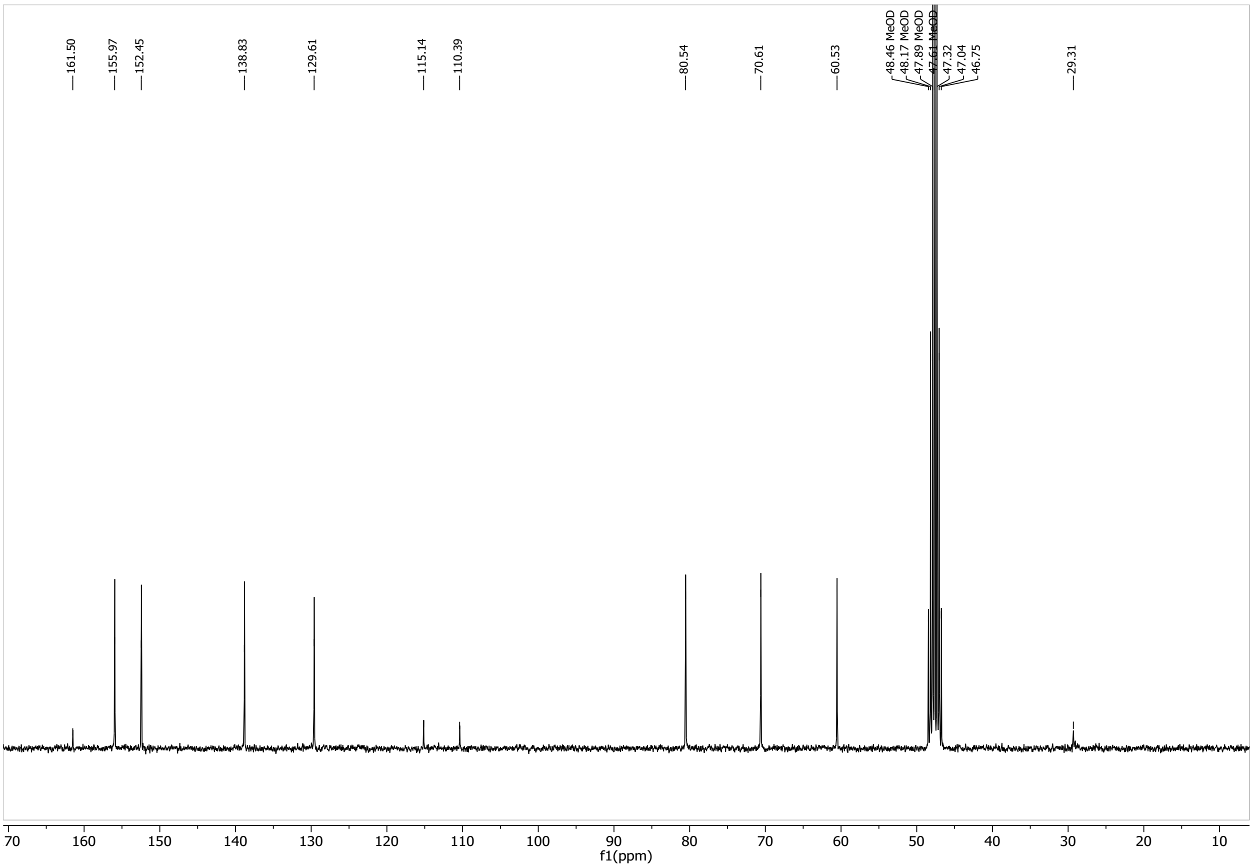

^{1}H-NMR spectrum (300 MHz, CD_3OD) of compound **16**

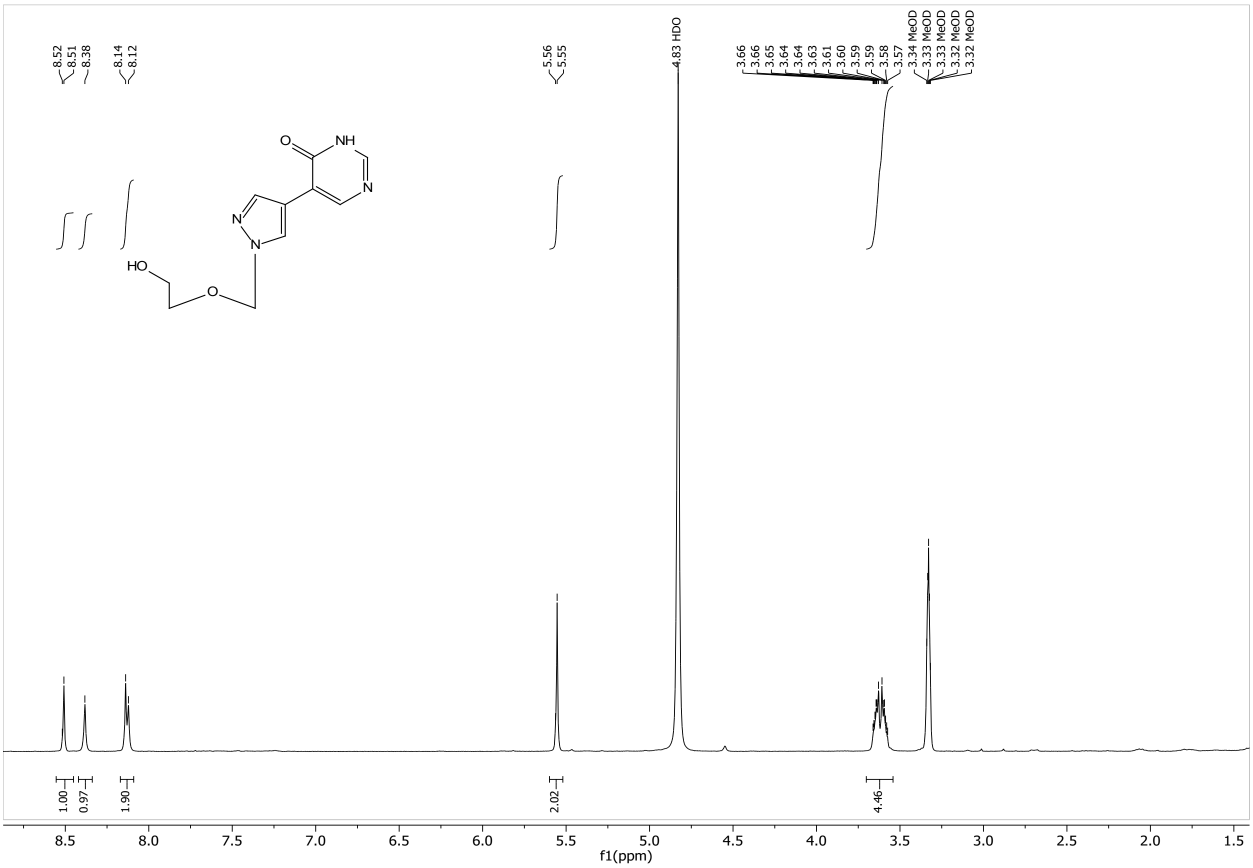

^{13}C-NMR spectrum (75.5 MHz, CD_3OD) of compound **16**

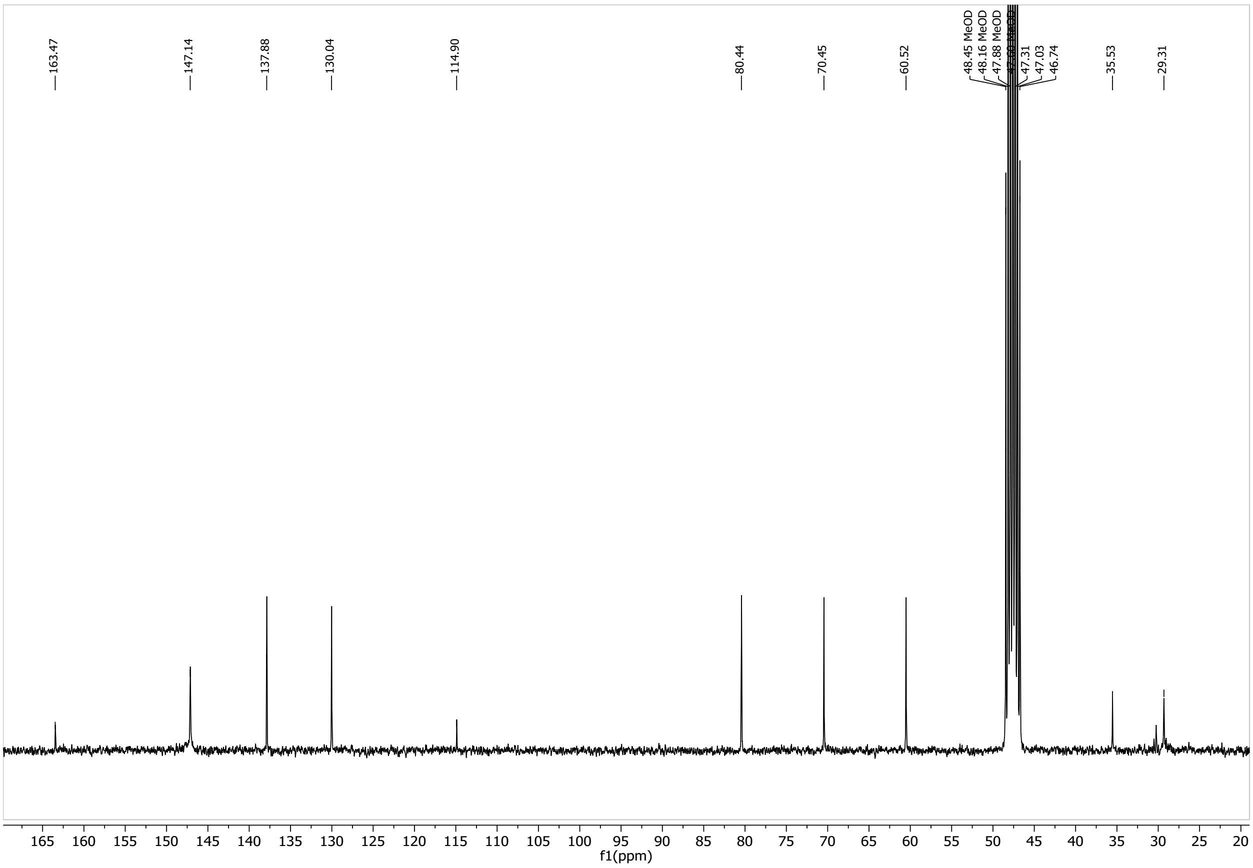

HRMS spectrum of compound **1**

Display Report

Analysis Info

Analysis Name	D:\Data\Chizhov\IMB\Matyugina\1\ln260_&clblow.d	Acquisition Date	25.01.2022 15:58:34
Method	tune_low_1550.m	Operator	BDAL@DE
Sample Name	1	Instrument / Ser#	maXis 43
Comment	CH3CN 100 %, dil. 200, calibrant added		

Acquisition Parameter

Source Type	ESI	Ion Polarity	Positive	Set Nebulizer	0.4 Bar
Focus	Not active			Set Dry Heater	180 °C
Scan Begin	50 m/z	Set Capillary	4500 V	Set Dry Gas	4.0 l/min
Scan End	1550 m/z	Set End Plate Offset	-500 V	Set Divert Valve	Source

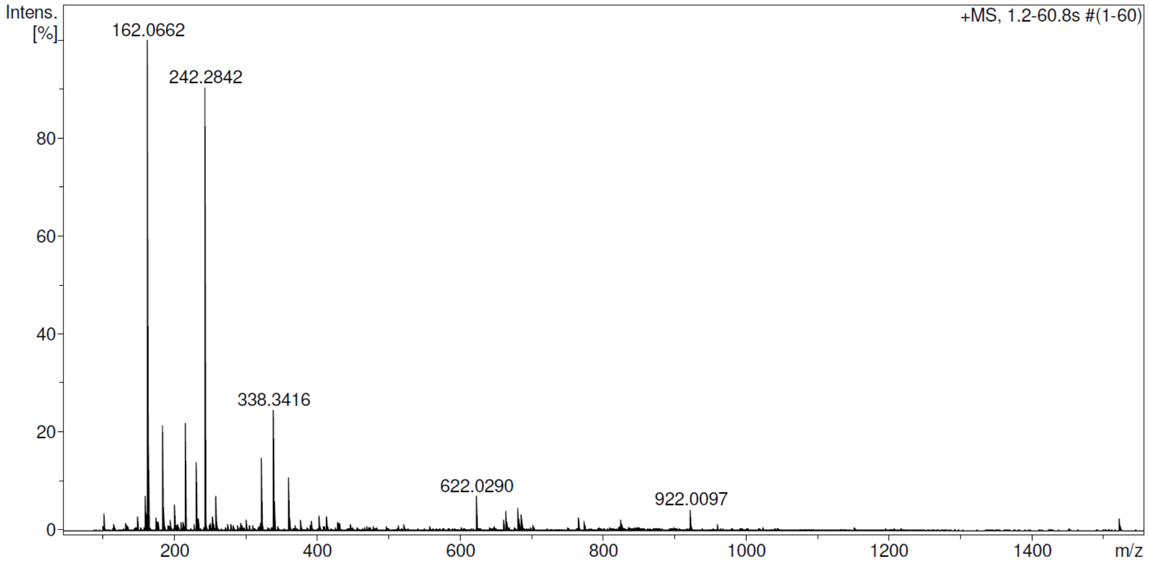

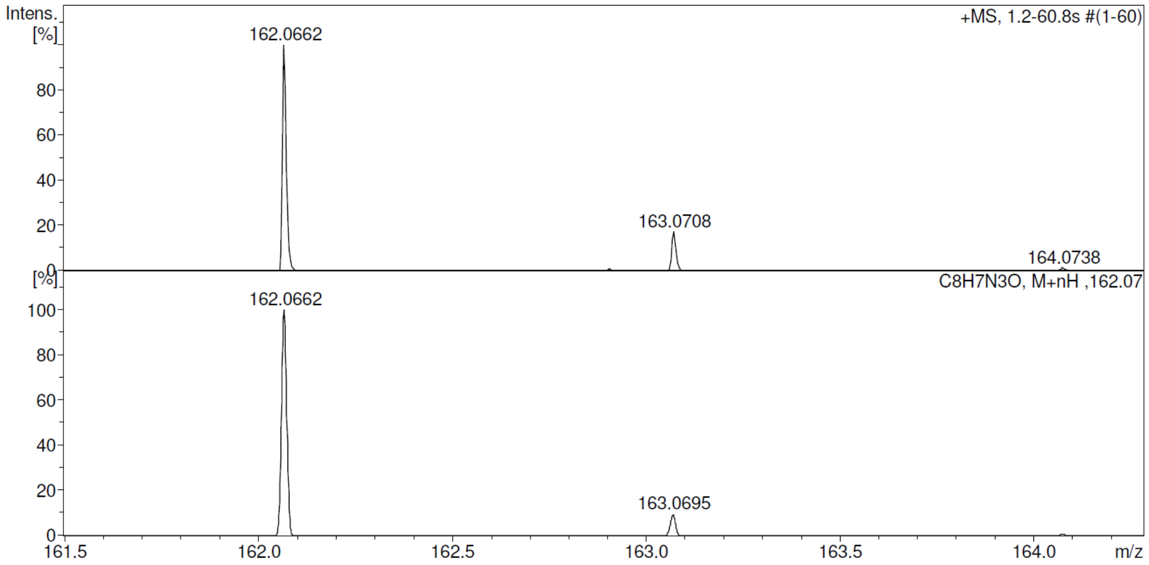

HRMS spectrum of compound **2**

Display Report

Analysis Info

Analysis Name	D:\Data\EM-121 (LN-56)_41_01_3936.d	Acquisition Date	12/27/2022 4:59:01 PM
Method	la_2.2_small.m	Operator	BDAL@DE
Sample Name	2	Instrument	compact 8255754.20088
Comment			

Acquisition Parameter

Source Type	ESI	Ion Polarity	Positive	Set Nebulizer	0.4 Bar
Focus	Active	Set Capillary	4500 V	Set Dry Heater	180 °C
Scan Begin	50 m/z	Set End Plate Offset	-500 V	Set Dry Gas	6.0 l/min
Scan End	3000 m/z	Set Charging Voltage	2000 V	Set Divert Valve	Source
		Set Corona	0 nA	Set APCI Heater	0 °C

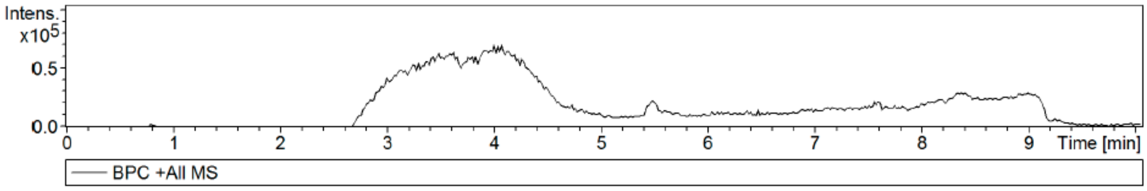

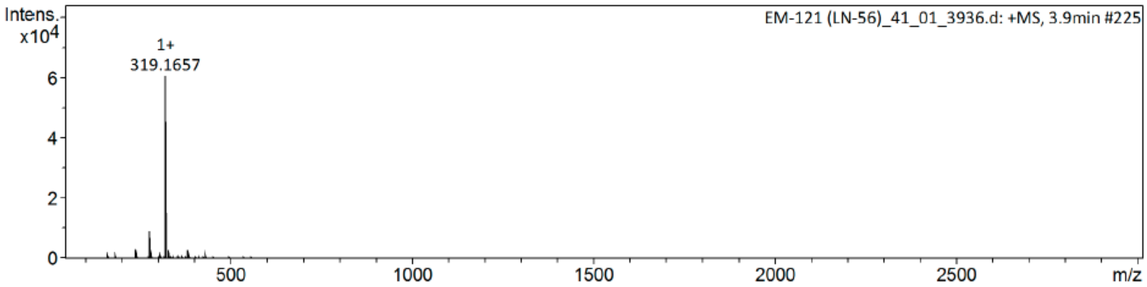

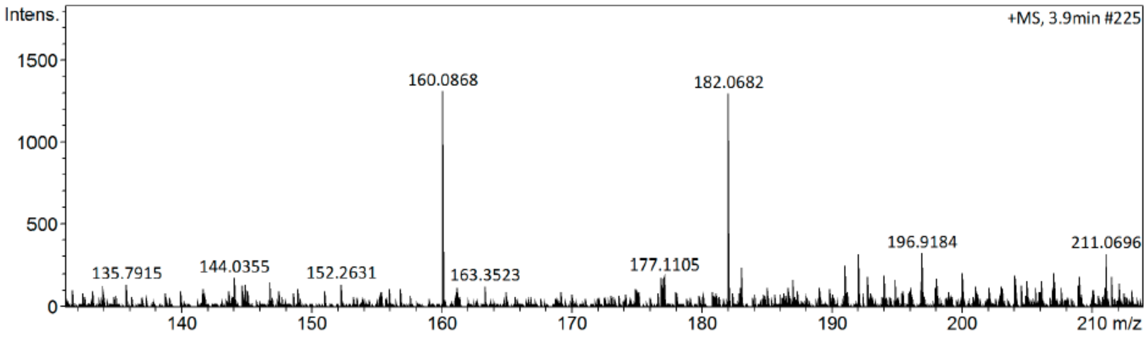

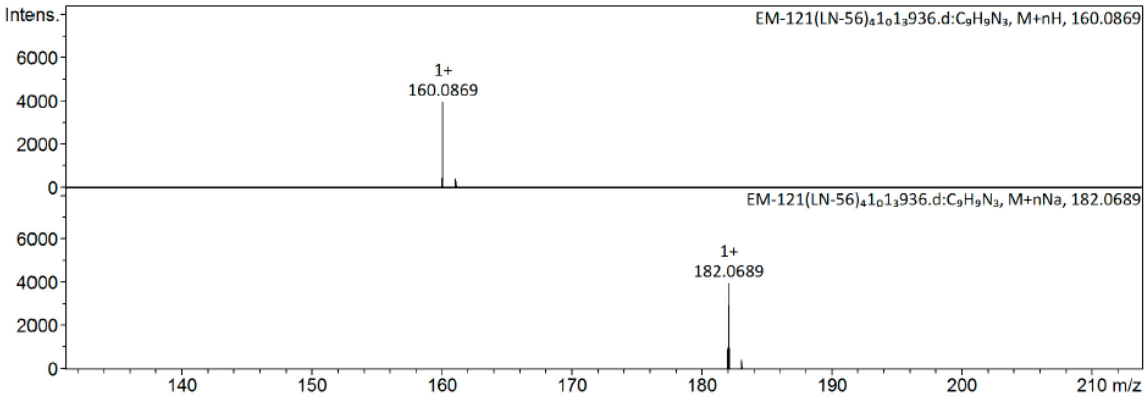

HRMS spectrum of compound **3**

Display Report

Analysis Info

Analysis Name	D:\Data\Chizhov\IMB\Matyugina\1\ln272_&clblow.d	Acquisition Date	25.01.2022 16:05:20
Method	tune_low_1550.m	Operator	BDAL@DE
Sample Name	3	Instrument / Ser#	maXis 43
Comment	CH3CN 100 %, dil. 2000, calibrant added		

Acquisition Parameter

Source Type	ESI	Ion Polarity	Positive	Set Nebulizer	0.4 Bar
Focus	Not active			Set Dry Heater	180 °C
Scan Begin	50 m/z	Set Capillary	4500 V	Set Dry Gas	4.0 l/min
Scan End	1550 m/z	Set End Plate Offset	-500 V	Set Divert Valve	Source

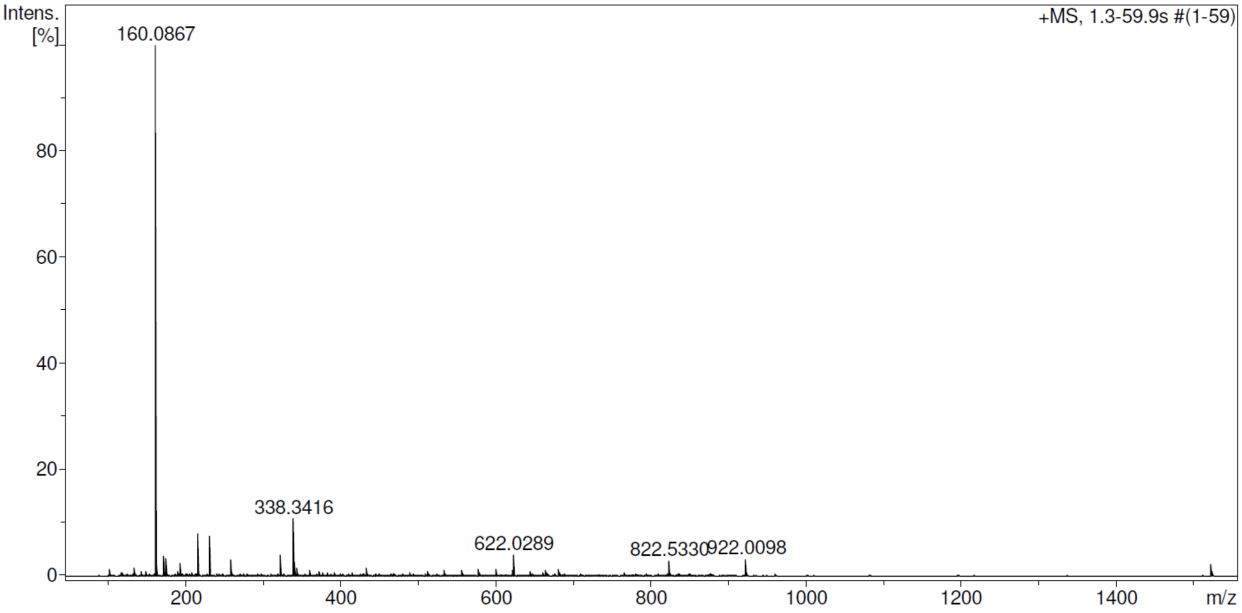

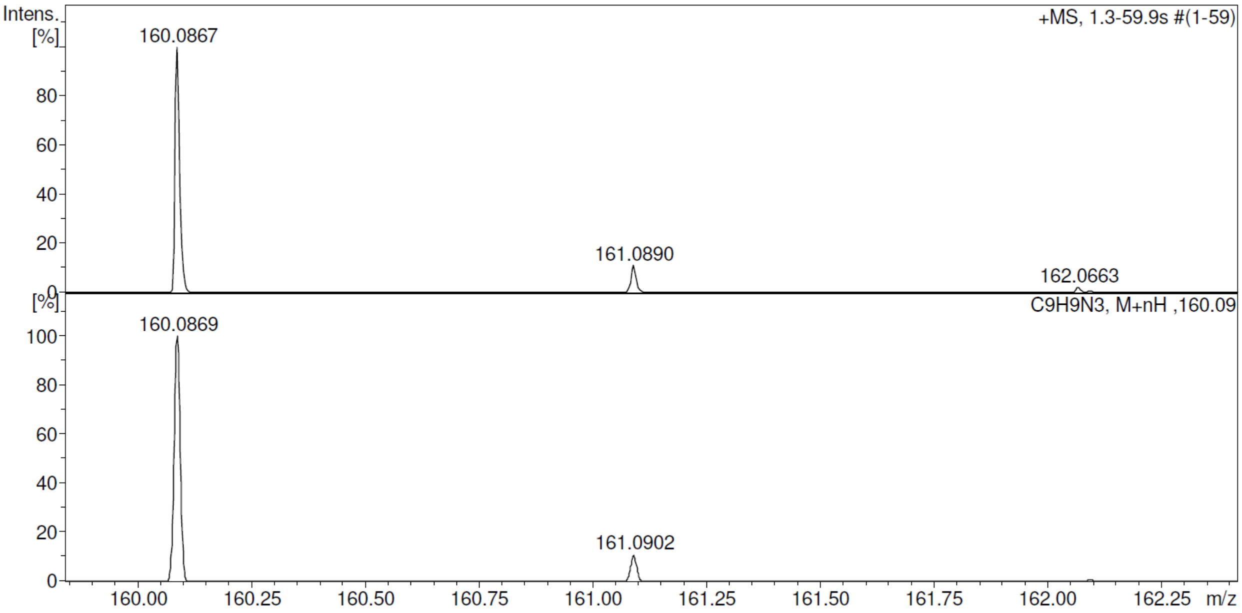

HRMS spectrum of compound **4**

Display Report

Analysis Info

Analysis Name	D:\Data\Chizhov\IMB\Matyugina\1\ln205_&clblow.d	Acquisition Date	25.01.2022 15:51:54
Method	tune_low_1550.m	Operator	BDAL@DE
Sample Name	4	Instrument / Ser#	maXis 43
Comment	CH3CN 100 %, dil. 2000, calibrant added		

Acquisition Parameter

Source Type	ESI	Ion Polarity	Positive	Set Nebulizer	0.4 Bar
Focus	Not active			Set Dry Heater	180 °C
Scan Begin	50 m/z	Set Capillary	4500 V	Set Dry Gas	4.0 l/min
Scan End	1550 m/z	Set End Plate Offset	-500 V	Set Divert Valve	Source

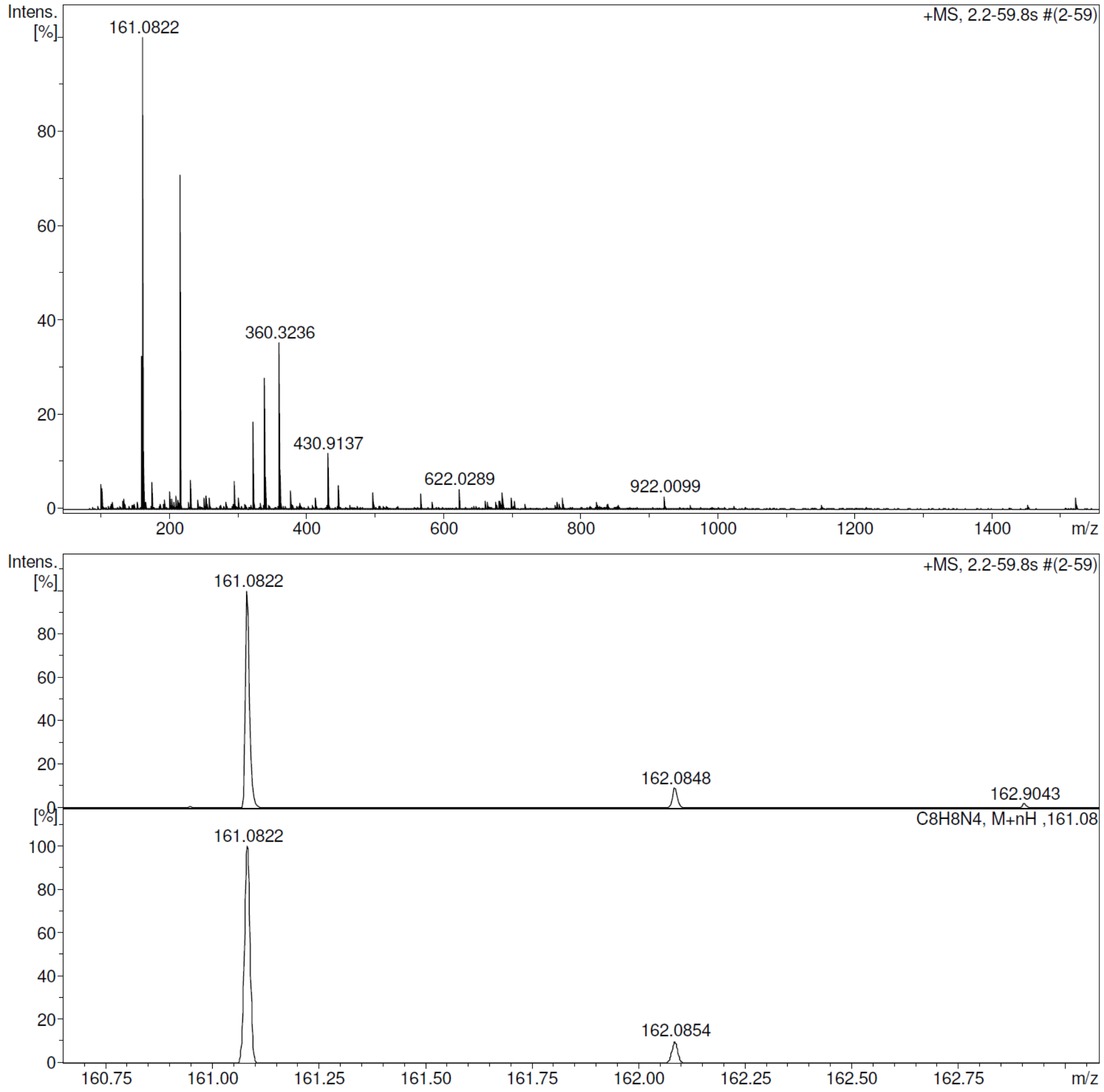

HRMS spectrum of compound **13**

Display Report

Analysis Info

Analysis Name	D:\Data\EN-116 (LN-293)_8_01_3908.d	Acquisition Date	11/30/2022 8:25:15 PM
Method	la_2.2_small.m	Operator	BDAL@DE
Sample Name	13	Instrument	compact 8255754.20088
Comment			

Acquisition Parameter

Source Type	ESI	Ion Polarity	Positive	Set Nebulizer	0.4 Bar
Focus	Active	Set Capillary	4500 V	Set Dry Heater	180 °C
Scan Begin	50 m/z	Set End Plate Offset	-500 V	Set Dry Gas	6.0 l/min
Scan End	3000 m/z	Set Charging Voltage	2000 V	Set Divert Valve	Source
		Set Corona	0 nA	Set APCI Heater	0 °C

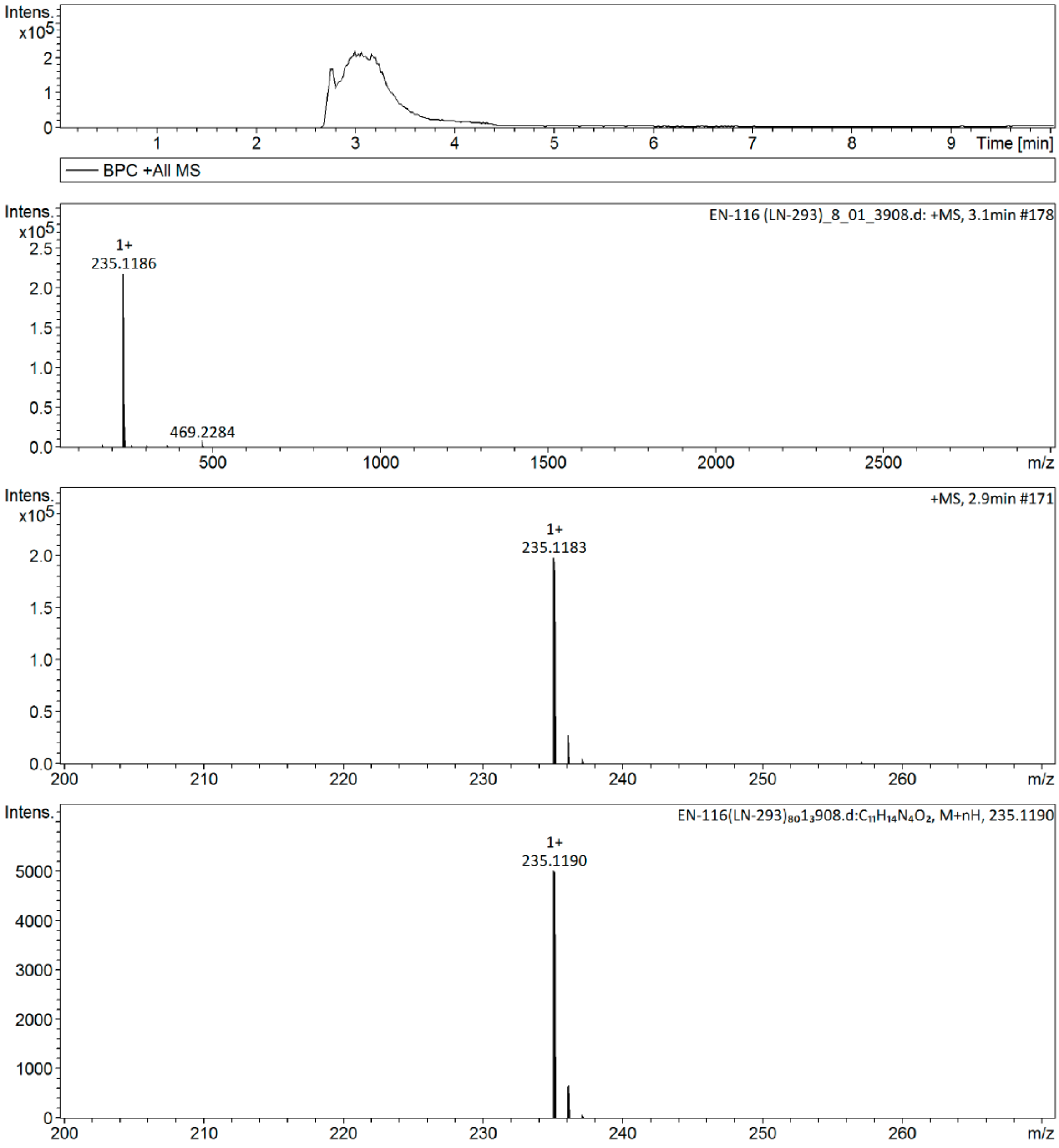

HRMS spectrum of compound **14**

Display Report

Analysis Info

Analysis Name	D:\Data\Chizhov\IMB\Matyugina\Oct_21_2022\In-266_&clblow.d	Acquisition Date	21.10.2022 14:12:12
Method	tune_low.m	Operator	BDAL@DE
Sample Name	14	Instrument / Ser#	micrOTOF CH3CN 10248
Comment	100 %, dil. 2000, calibrant added		

Acquisition Parameter

Source Type	ESI	Ion Polarity	Positive	Set Nebulizer	0.4 Bar
Focus	Not active			Set Dry Heater	180 °C
Scan Begin	50 m/z	Set Capillary	4500 V	Set Dry Gas	4.0 l/min
Scan End	3000 m/z	Set End Plate Offset	-500 V	Set Divert Valve	Waste

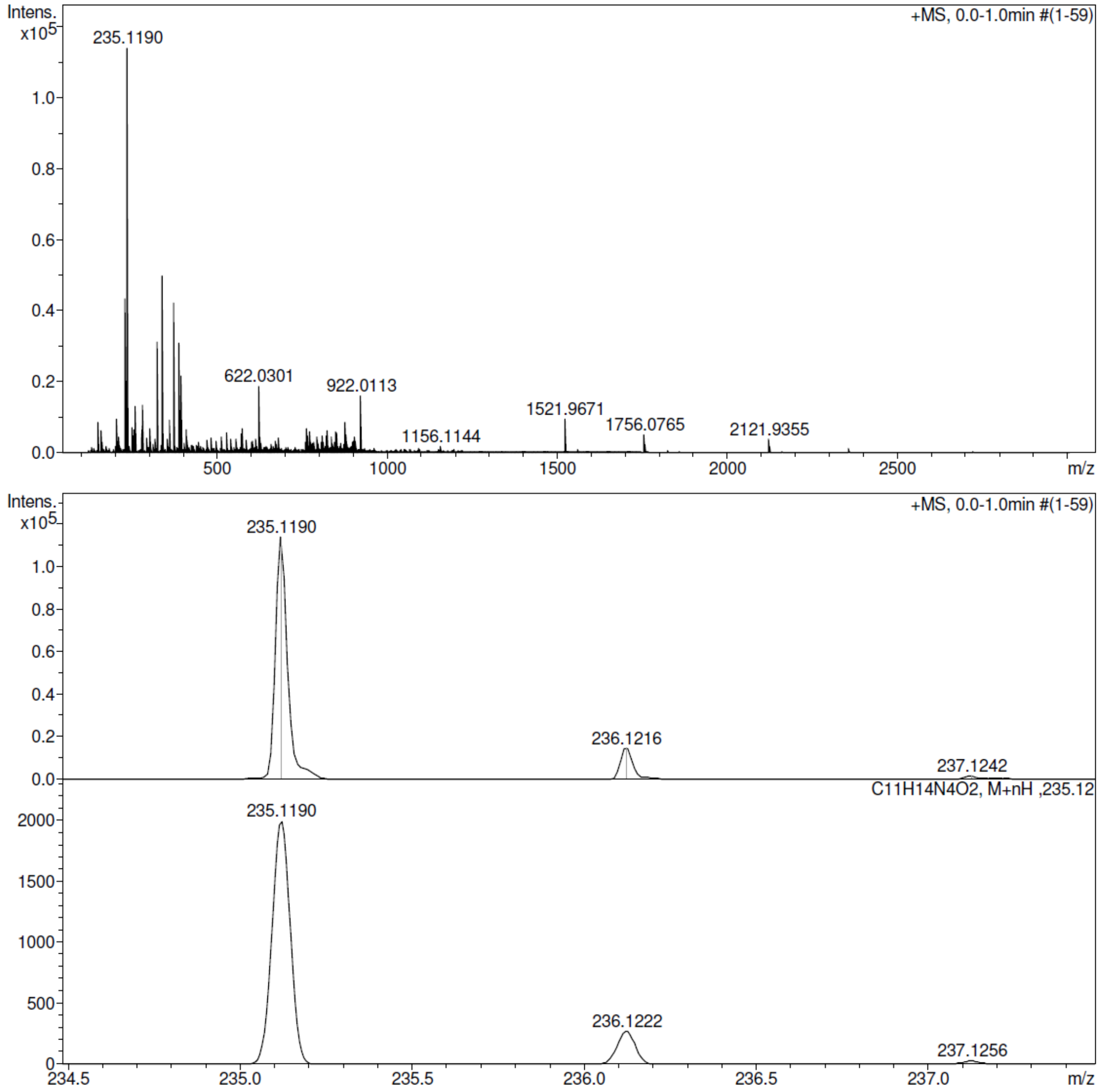

HRMS spectrum of compound **15**

Display Report

Analysis Info

		Acquisition Date	21.10.2022 14:16:23
Analysis Name	D:\Data\Chizhov\IMB\Matyugina\Oct_21_2022\In-268_&clblow.d		
Method	tune_low.m	Operator	BDAL@DE
Sample Name	15	Instrument / Ser#	micrOTOF CH3CN 10248
Comment	100 %, dil. 2000, calibrant added		

Acquisition Parameter

Source Type	ESI	Ion Polarity	Positive	Set Nebulizer	0.4 Bar
Focus	Not active			Set Dry Heater	180 °C
Scan Begin	50 m/z	Set Capillary	4500 V	Set Dry Gas	4.0 l/min
Scan End	3000 m/z	Set End Plate Offset	-500 V	Set Divert Valve	Waste

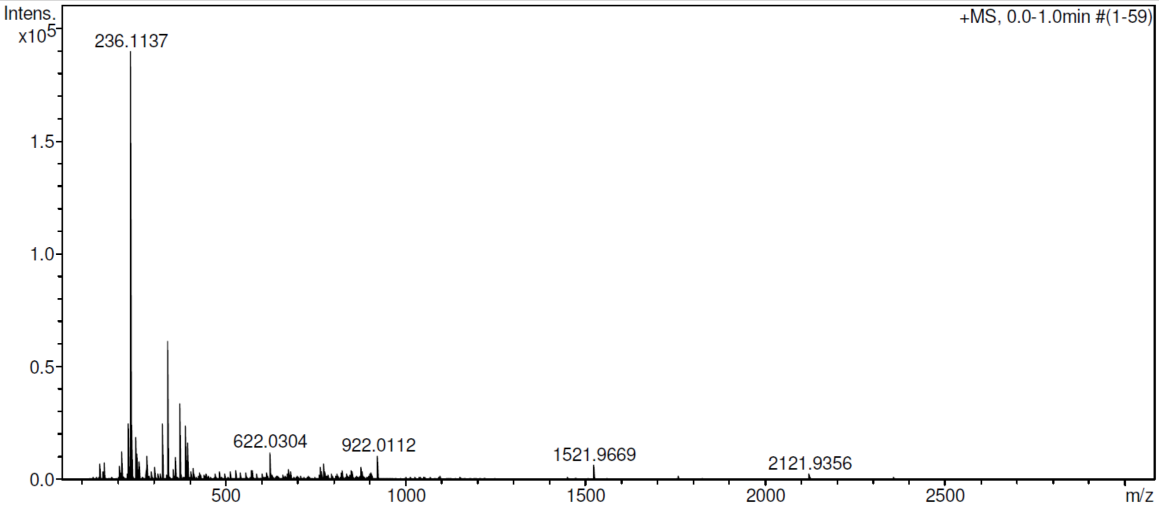

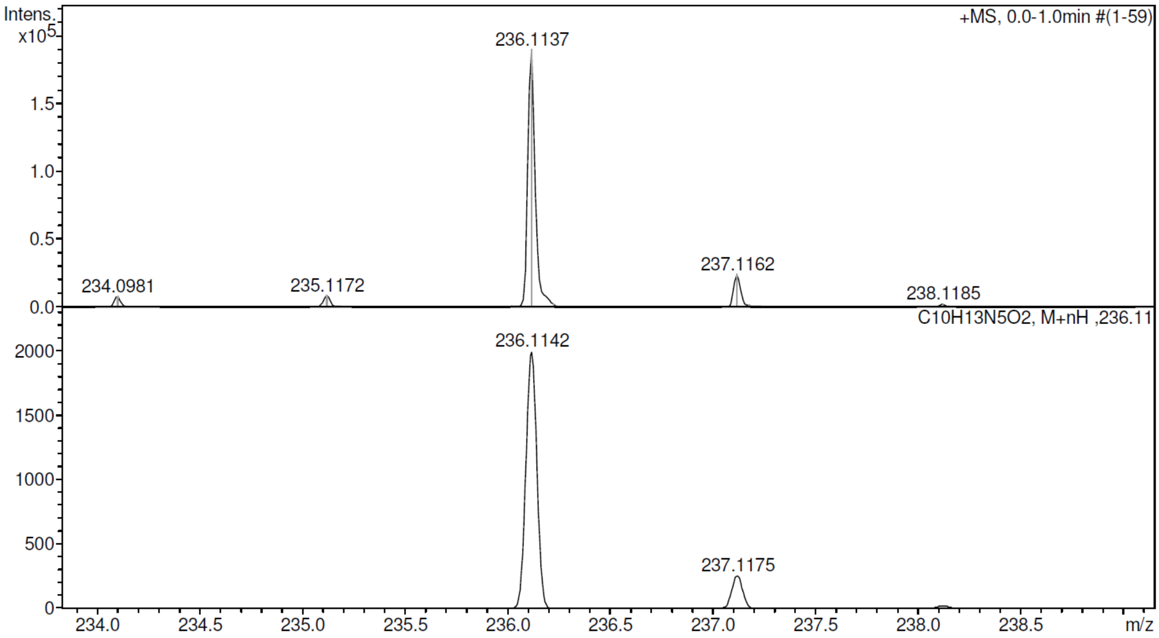

HRMS spectrum of compound **16**

Display Report

Analysis Info

Analysis Name	D:\Data\Chizhov\IMB\Matyugina\Oct_21_2022\In-300_&clblow.d	Acquisition Date	21.10.2022 14:24:08
Method	tune_low.m	Operator	BDAL@DE
Sample Name	16	Instrument / Ser#	micrOTOF CH3CN 10248
Comment	100 %, dil. 200, calibrant added		

Acquisition Parameter

Source Type	ESI	Ion Polarity	Positive	Set Nebulizer	0.4 Bar
Focus	Not active			Set Dry Heater	180 °C
Scan Begin	50 m/z	Set Capillary	4500 V	Set Dry Gas	4.0 l/min
Scan End	3000 m/z	Set End Plate Offset	-500 V	Set Divert Valve	Waste

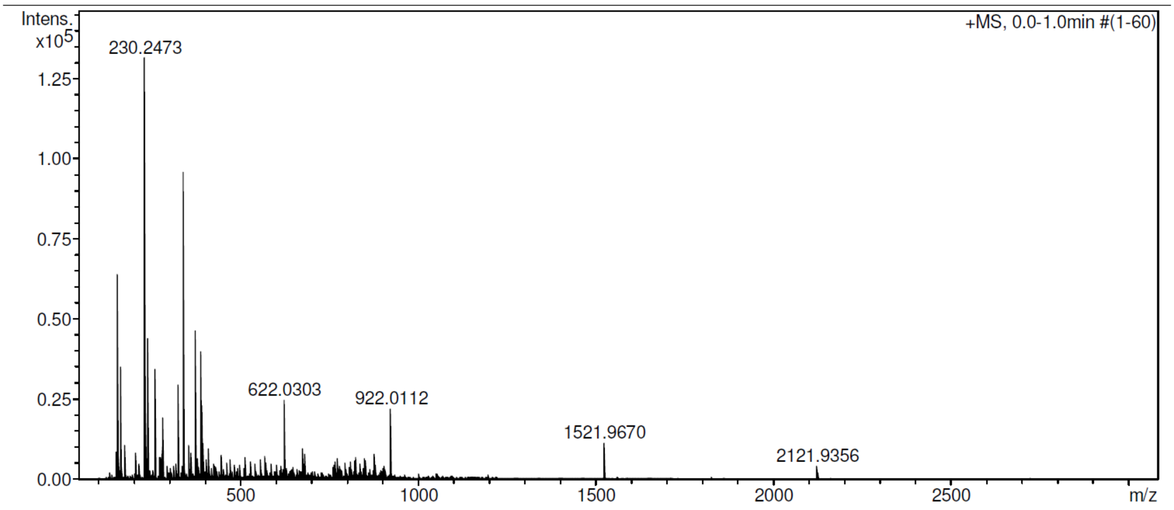

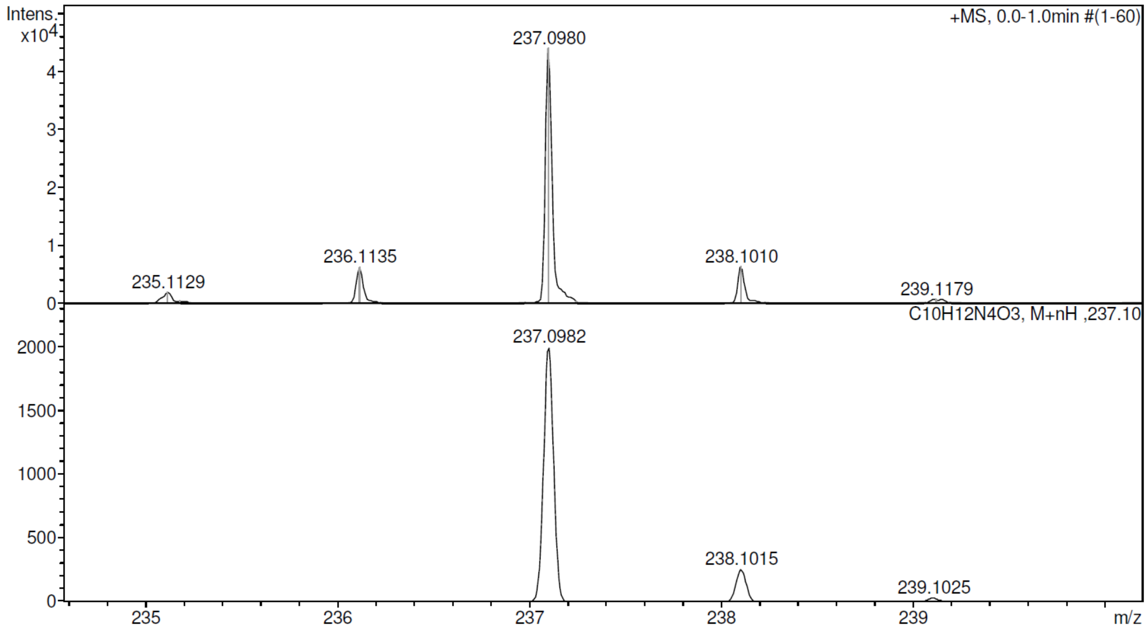

Figure S1. Lineweaver–Burk plots for the inosine phosphorolysis at concentrations of compounds 1, 2, 3 and 4 equal to 0, 0.5, 1 and 2 mM. (A) compound 1, (B) compound 2, (C) compound 3, (D) compound 4.

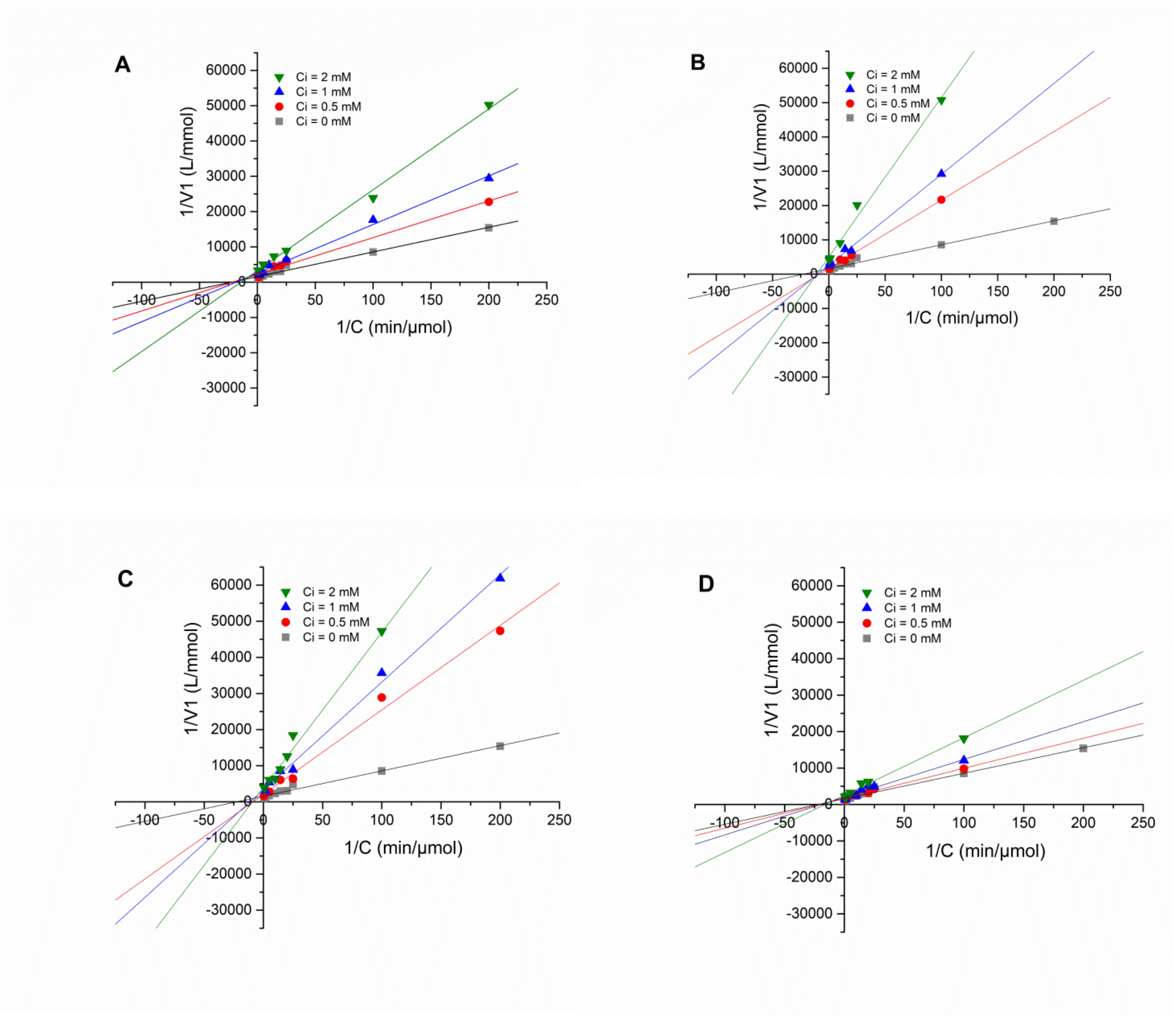

Figure S2. 2D diagrams of complexes PNP with various type of ligands.

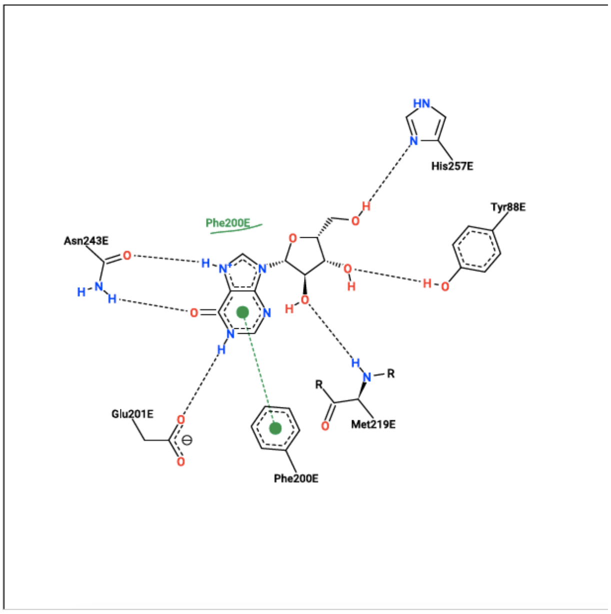

Complex with inosine (PDB ID code 1RCT)

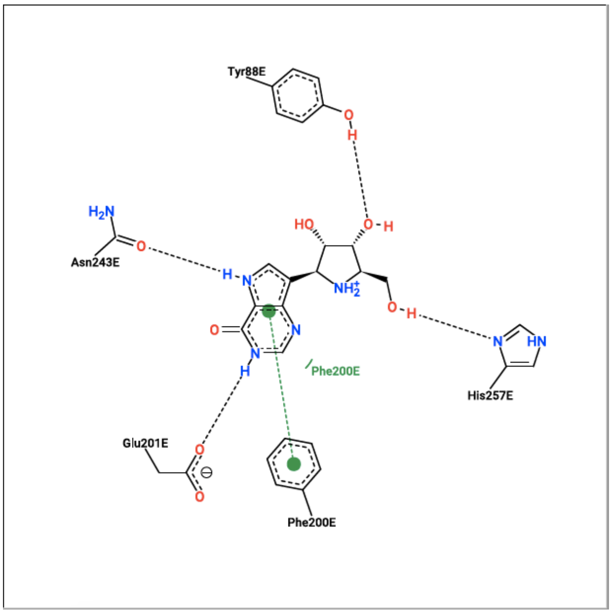

Complex with Immucillin H (PDB ID code 1PF7)

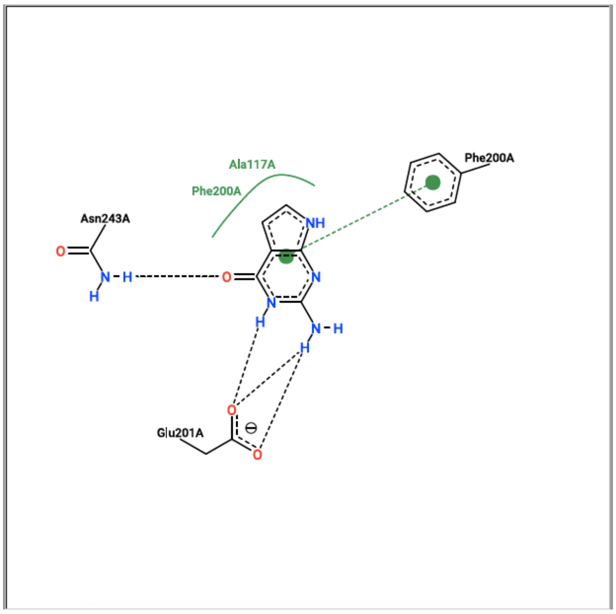

Complex with 7-deazaguanine. (PDB ID code 3INY)

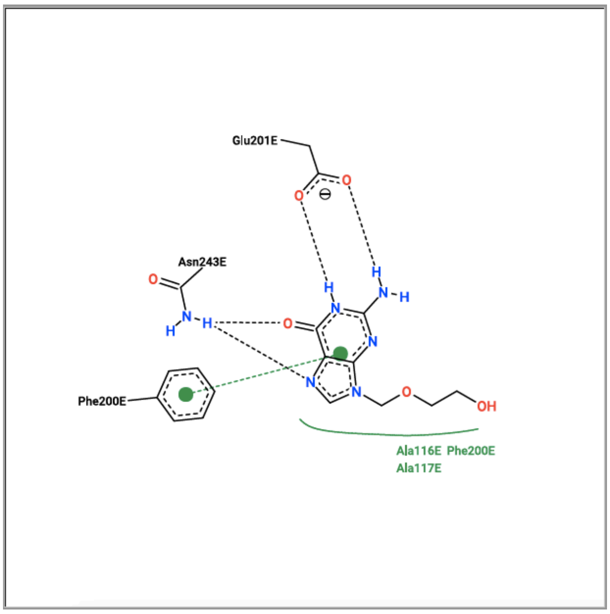

Complex with acyclovir (PDB ID code 1PWY)

Figure S3. Structure of the 3INY with re-docked 7-deazaguanine (An overlap the red wire – native ligand from PDB database; the red stick docked ligand. The box for docking ligands was highlighted in green (12 Å × 10 Å × 10 Å.)

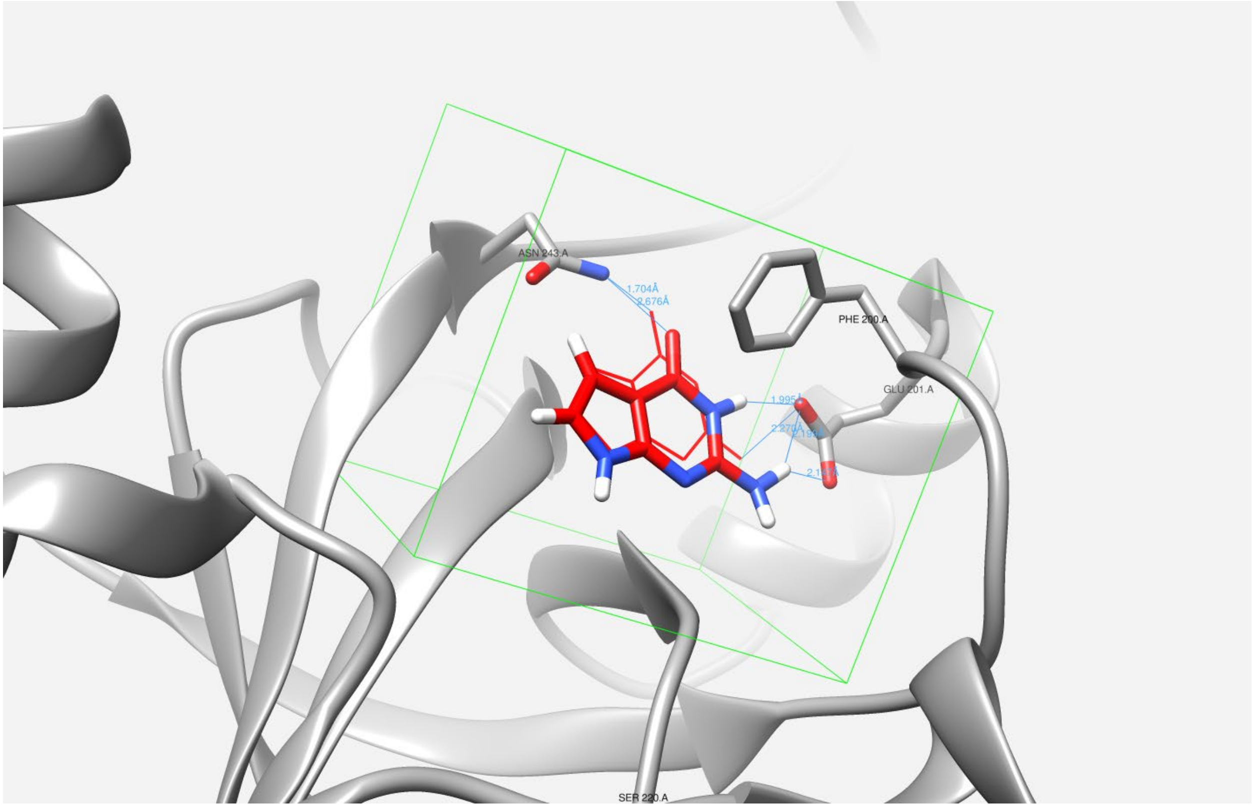

Supplement: Supplementary file 1 [file molecules-28-00928-s001.zip › molecules-2152156-supplementary.pdf]
